# Guest-ant social parasites avoid conflict with social hosts using venom signaling

**DOI:** 10.1371/journal.pone.0345143

**Published:** 2026-08-12

**Authors:** Matthew R. Boot, Kyle S. Sozanski, Mazie Davis, Ian M. Hamilton, Rachelle M. M. Adams

**Affiliations:** 1 Department of Evolution, Ecology and Organismal Biology, The Ohio State University, Columbus, Ohio, United States of America; 2 Tiffin University, Tiffin, Ohio, United States of America; 3 Department of Mathematics, The Ohio State University, Columbus, Ohio, United States of America; 4 Smithsonian Tropical Research Institute, Smithsonian Institution, Ancon, Panamá, República de Panamá, United States of America; Universidade de São paulo, BRAZIL

## Abstract

Communication is a fundamental component of symbiosis but when the interests of sender and receiver are not aligned, manipulative rather than informative signaling, may occur between species. Although honest parasite signals are rarely explored, they could be favored if the host-parasite relationship provides an opportunity for a reduction of mutual costs or an acquisition of mutual benefits. Eusocial insect societies exhibit a unique type of symbiotic lifestyle—parasitism of one social unit (i.e., colony) by another. These social parasites gain resources despite risking repeated, and potentially costly, interactions with hosts. *Megalomyrmex symmetochus* “guest ant” parasites cohabit, long-term, within the nest of a single colony of *Sericomyrmex amabilis*, a fungus-farming ant. Although *M. symmetochus* exploit their stingless hosts for resources, they directly rely on their host colony for survival and reproduction, and thus, have been demonstrated to protect their host colony from threats to the shared nest using venom weaponry. We use behavioral observation of staged host-parasite conflict, lethal-dose assays, and direct venom measurements to show that *M. symmetochus* uses conspicuous and costly displays of venom during interactions with hosts. *Megalomyrmex symmetochus* is observed to dispense alkaloid venom directly through stinging, but more frequently, indirectly through airborne venom dispersal of volatile pyrrolizidine alkaloids. We further demonstrate that indirect venom use can alter interaction outcomes if hosts switch to non-aggressive behavioral tactics. We interpret this as an honest signal of the threat of envenomation, which reduces the risk of lethal aggression between heterospecific nestmates. We argue that venom signaling in this system, from parasites to their hosts, benefits both species due to the alignment of interest resulting from two different forms of conditional dependence 1) parasite reliance on host colony persistence and 2) the potential role that the parasites play in defending resources of the host colony. This work also demonstrates that communication strategies to manage conflict can arise in antagonistic partnerships and helps us understand how groups with competing interests are able to coexist.

## Introduction

In animal communication systems, signals are evolved structures or actions that elicit a response from a receiver [1]. In order for an honest signal and response to be favored by natural selection, the response must, on average, benefit both senders and receivers [2]. The mutual benefit of signaling systems is often clear in cooperative interactions; however, signaling is also frequent in other types of animal interactions. For example, in competitive interactions (e.g., vocal calling in male frogs; [3]), signals can allow senders and receivers to reduce costs associated with aggression [4]. In predator-prey systems, signaling by prey (e.g., flash of a white deer tail; [5]) can benefit both the predator and their potential prey by circumventing costly pursuit [6,7]. In these non-cooperative systems, signals reduce mutual costs associated with the antagonistic relationship.

Similarly, parasites stand to gain by exploiting their hosts; either manipulating, or else, avoiding detection by hosts [8–10]. Examples of honest parasite signaling within the animal kingdom, however, have rarely been explored ([9,11]; but see [12]). Still, honest parasite signaling to hosts could, in theory, be favored if the host-parasite relationship provided an opportunity for a reduction of mutual costs or an acquisition of mutual benefits.

In ants, social parasitism occurs when one colony exploits the communal resources (e.g., food, brood, workforce, nest space) of another ant colony [13,14]. Within the shared social environment, parasitic individuals can incur significant costs—each individual risks repeated interactions with hosts and is subject to detection and coordinated aggression by hosts as a collective force [15]. As a result of the antagonistic social environment, parasites of social species have evolved traits or behavioral tactics that allow individuals to overcome collective host defenses. In particular, social parasites are known to use an array of chemical mimicry, propaganda, or appeasement tactics to avoid or subvert these host defenses [13,14,16,17]. “Guest ants” in the genus *Megalomyrmex* (Forel, 1885, Formicidae: Myrmicinae: Solenopsidini), however, specialize in the exploitation of stingless fungus-growing ants (Formicidae: Attini: Attina) in a direct and conspicuous manner using alkaloid venom, a form of chemical weaponry, rather than stealth or chemical deceit [18–21].

*Megalomyrmex* guest ants permanently cohabit within the host colony’s nest and are completely reliant on the host colony to complete their lifecycle. Parasite foundresses enter the host colony during its founding stage, building their colony as the host colony grows. The parasite colony subsists entirely off host resources (fungal garden, brood; [21]). As a result, there are predicted costs of escalated conflict for both symbionts. Host worker mortality can reduce host colony size, and thereby, host reproductive output [22–24]. On the other hand, despite their venom weaponry, the fitness of the parasite colony is also ultimately constrained by their host colony’s size and productivity. As a result, guest ants may face long-term costs if they limit host colony growth too severely (through lethal venom use) and could instead benefit from exploiting their hosts prudently [25].

*Megalomyrmex symmetochus* guest ants have even been found to engage in an unusual context-dependent mutualism with their host ants, *Sericomyrmex amabilis* [26]. While *S. amabilis* are stingless, they still protect nest resources using their mandibles to mutilate intruders; *M. symmetochus* workers have been observed missing legs or antennae in the field and lab [27]. Despite this host-parasite antagonism, *M. symmetochus* will defend their host colony from within (using venom stinging) against nest raids by a third ant species, *Gnamptogenys hartmani* (their only known natural enemy)*,* which otherwise decimates the host fungus garden [26]. This guest ant relationship is context-dependent because guest ants consume host brood and garden, but the overall fitness impact on the host colony can shift depending on the threat level of *G. hartmani* in a given population.

Notably, stinging of *S. amabilis* hosts by *M. symmetochus* and other forms of direct conflict between the two species is not often observed in shared nests [20,26–28]. Instead, *M. symmetochus* have been observed in the lab to engage in conspicuous “gaster-flagging” behaviors, where venom is extruded from the sting and volatilized when interacting with host workers (as in, [19]). Gaster flagging has been described in several other species in the Solenopsidini tribe and is exhibited by various species of Hymenoptera in colony defense and interference competition [19,29–32].

We hypothesized that *M. symmetochus* not only uses venom directly against hosts (i.e., stinging) but also uses it indirectly (via airborne volatilization) as a social signal in ways that reduce costly host-parasite conflict. Guest ant venom could provide hosts with information about parasite fighting ability and affective state (willingness/motivation to escalate) allowing hosts to assess conflict risk [2], similar to other hymenopteran venoms that have communicative function [32,33].

The main objective of this study was to understand the role of the putative signal used during host-parasite conflict. To accomplish this, we staged artificial host-parasite pairings of lab-maintained colonies. We predicted that parasite venom use, both direct and indirect, would increase submissive behavior by hosts. To test this, we assessed 1) overall venom use by parasites, 2) the conditions under which interactions arise and escalate to direct aggression, 3) when parasites use venom, and the effects of venom use on host behavior, and 4) how interactions terminate (at the group level). We then explored limitations to parasite venom-use imposed by an individual’s venom storage and the amount of venom needed to kill host workers.

## Materials and methods

### Specimen collection and rearing

Colonies of *Sericomyrmex amabilis* and *Megalomyrmex symmetochus* were excavated and collected from Parque Nacional Soberanía along Plantation Road and Pipeline Road near Gamboa, Panama (9.1189, −79.7030) or on Barro Colorado Island (9.1659, −79.8366) from 2011 to 2022. Colonies were kept inside temporary plastic enclosures until transported back to either The University of Copenhagen, Denmark, or The Ohio State University's Museum of Biological Diversity, USA, where colonies were moved into plaster-lined permanent nest enclosures and kept at approximately 23–25° C. In both cases, colonies were regularly checked and resupplied with water (to maintain internal humidity) and food (cornmeal, oatmeal, oak catkin) ad libitum, and waste was removed when needed [34].

*Megalomyrmex symmetochus* is listed as “vulnerable” by the International Union for Conservation of Nature [35], therefore whole colony collections were avoided whenever possible. Permits for the collection of *S. amabilis* and *M. symmetochus* on public land were obtained from Panama's Ministerio de Ambiente (Autoridad Nacional del Ambiente until 2015) under permit numbers SE/A-24–11, SE/A-29–12, SE/A-41–13, SE/A44-19, SE/A-27–2020. Our field sites include colonies in a long-term study where we assess the lifetime of parasitized and non-parasitized colonies. We therefore go to great lengths to avoid collecting queens and take only a subset of the colony members and fungal garden for our work. Queenright colonies and queenless worker subsets collected with a portion of garden can be maintained under the laboratory conditions noted above for years [34]. This has enabled us to use the same colonies for multiple experiments and studies.

Ethical approval was not sought for the present study because animal research on invertebrates is not regulated. Institutional Animal Care and Use Committee guidelines were followed where possible; animal care was performed to a high standard, and humane endpoints were administered (using chilled 98% ethanol) for unresponsive individuals at the end of the toxicity assays to minimize potential pain and distress. Responsive ants used in the control or low dose treatments were returned to colony after all assays were completed. To investigate the behaviour of host ants against parasite ants, it was necessary to conduct staged experiments. We restricted each trial to 5 minutes, and host ants were euthanized in chilled 98% ethanol. Because of their change in chemical profile due to alkaloid venom exposure, they may be attacked by their sisters if returned to their home colony. Parasite workers were returned to their laboratory nest following each experiment.

### Behavioral interactions

We reanalyzed videos of staged host-parasite encounters from Neupert *et al*. (2018), focusing on a subset of videos (n = 51), pairing *S. amabilis* hosts from non-parasitized colonies with foreign *M. symmetochus* parasites. These pairings consistently generated direct interspecific conflict for scoring [20]. The videos were selected because they contained clear, direct interactions between the host and parasite species, which is generally reduced in naturally parasitized colonies [20]. Our dataset represents a high-conflict social environment for parasites, where the staged encounters are meant to simulate aggressive attacks by native hosts to their own parasite workers. We have observed interspecific conflict between *S. amabilis* and *M. symmetochus* in the lab. For example, conflict has often been observed when the two species are producing reproductives. Host workers have been observed attacking *M. symmetochus* parasites and parasite workers have been observed chewing the wings off of host alates [27]. Conflict can also occur between workers of both species, particularly when the host fungal garden appears unhealthy (*MRB personal observation*).

Depending on the year, *M. symmetochus* can be difficult to locate in the wild (*RMMA personal observation*). Due to the small number of parasite colonies available for experimentation, each parasite colony was paired against multiple host colonies for observational video recordings. A total of five host colonies and six parasite colonies were evaluated across the 51 videos examined (Supplement S1 Appendix). Each observational pairing contained five hosts and one parasite which were scored at the group level, this simulates real nest conditions where parasites are outnumbered numerically by hosts by at least 2:1 [24,36]

### Focal behaviors and ethogram

To characterize host-parasite conflict, we adapted and expanded the *S. amabilis* ethogram from Neupert et al., 2018 to include the behaviors of the parasite, *M. symmetochus* (Table 1). We included behaviors relevant to conflict interactions between the host and parasite, and also assessed behaviors according to their functional role within an interaction (Table 1). We defined conflict as any behavior that can act as direct tactile aggression or venom dispersion and the set of evasive/postural responses to these stimuli. We binned this set of “conflict behaviors” into one of several behavioral categories: aggressive or submissive for *S. amabilis* hosts, direct or indirect alkaloid dispensing for *M. symmetochus*. Additional behaviors that were generally associated with host-parasite interaction, but which occurred both inside and outside of direct conflict (e.g., “conflict-related”) were scored as “other” (Table 1).

**Table 1:** Ethogram table of focal behaviors based off of Neupert *et al.*, 2018.

Behavior		Description	Behavioral type	Behavioral category
**Host (*S. amabilis*)**				
	inspection	Focal ant's antennae are extended and held aloft from the ground to sense air currents	arousal	*other*
	rush	Focal ant runs quickly in forward direction, > 1 body length	arousal/offensive	aggressive
	charge/lunge	Focal ant very rapidly moves forward < 1 body length, usually preceding bite	offensive*	aggressive
	bite	Focal ant bites at or on the stimulus ant	offensive*	aggressive
	pull	Focal ant pulls stimulus ant during bite (often from appendages)	offensive*	aggressive
	carry	Focal ant lifts/carries the stimulus ant during bite	offensive*	aggressive
	back up	Focal ant steps backward 3 or more steps within stimulus ant vicinity	evasive/avoidant	submissive
	turn	Focal ant rapidly pivots (>90 degrees) after close contact with stimulus ant; may co-occur with other defensive postures, angle relaxed to >45 if co-occurring with other defensive/evasive postures	evasive/avoidant	submissive
	antenna tuck	Focal ant retracts antennae (>80% retraction) and positions head downward	defensive	submissive
**Parasite (*M. symmetochus*)**				
	inspection	Focal ant's antennae are extended and held aloft from the ground to sense air currents	arousal	*other*
	bite	Focal ant bites at or on the stimulus ant	offensive*	aggressive
	raised gaster	Focal ant raises abdomen, and/or holds abdomen raised	signal	venom – indirect
	gaster flag	Focal ant flexes abdomen rythmically, anteriodorsally, while in raised position	signal	venom – indirect
	side-swipe sting	Focal ant rapidly swipes abdomen sideways, off body axis, at stimulus ant while in close proximity (may occur along a horizontal or vertical axis)	offensive*/evasive	venom – direct
	gaster-tuck sting	Focal ant pokes/wipes abdomen tip between its legs at stimulus ant while in close proximity (usually during grappling)	offensive*	venom – direct

Behaviors were chosen for their relevance to host/parasite conflict during interactions. Specifically, focal conflict behaviors putatively characterize host or parasite arousal, avoidance, offense, and/or defense behaviors, as well as signaling behaviors. Offensive behaviors represent a potential stimulus for the heterospecific ant. Offensive behaviors marked with an asterisk are “direct” offensive-type behaviors, denoting unambiguous stimulus and potential for direct harm to the paired, non-focal individual. Note, venom is not always visible during venom-use behaviors, but is inferred from postural and movement cues.

Venom itself was, generally, not directly visible in our video recordings and is thus inferred based on the presence of the behaviors described in our ethogram. During observations of host-parasite conflict interactions, we identified two prominent alkaloid dispensing behaviors which involved contactless, indirect venom exposure: “raised-gaster” behavior where parasites simply raise their abdomen and hold this posture, and “gaster-flagging” behavior where parasites vigorously bend the raised gaster, flexing the tip rhythmically. Direct stinging behavior was also observed and divided into two types, gaster-tuck sting and side-swipe sting (Table 1).

### Video scoring

In each behavioral trial, five *S. amabilis* ants were selected haphazardly from a colony and placed in a petri dish (35 mm) with a piece of their fungus garden (ca. 100 mm^3^) and allowed to acclimate for 15 minutes. This setup was constructed to simulate the nest environment of the host, to promote natural behaviors between the ant test subjects. After 15 minutes, a single *M. symmetochus* ant was added to the petri dish and the entire arena was recorded for 5 minutes [20].

Using video playback, the focal behaviors of each ant in the dish engaging in an active interaction were visually scored and recorded in Microsoft Excel. All videos were scored by a single scorer. An interaction was defined as each instance in which the focal parasite ant was within two body-lengths of a host and either ant performed a conflict-related behavior. The proximity threshold was assessed visually. To avoid any effects of heterospecific interaction between ants that could carry over to subsequent interactions in an arena trial (including prior aggression, chemical cues, or venom-use), we scored only the first naïve interaction in each arena trial.

It is important to note that there are five hosts to one parasite in each arena trial. As a result, interactions with parasites can include multiple hosts (which is common in nest interactions). All behavioral analyses were performed at the group level; however, we recorded the identity of each interacting ant throughout for follow-up analyses of pairwise effects between the parasite and separate hosts within group interactions.

An interaction was considered to begin when any ant approached a heterospecific ant and demonstrated a conflict-related behavior within two body-lengths of the heterospecific. Conflict-related behavior that initiated prior to entering the range but continued into the proximity threshold was also scored. Behavioral scoring continued as long as focal ants remained in the vicinity of a heterospecific ant and used conflict-related behaviors. When conflict-related behaviors continued outside the proximity threshold, behaviors initiated before exiting were scored until those behaviors ended, and continuation of additional behaviors were noted.

Interactions were considered terminated when 1) both actors ceased conflict behaviors completely, or 2) *either actor* exhibited a cessation of conflict behaviors *while* exiting from the proximity threshold or traversing a distance greater than four body-lengths. When interactions ended, we also scored the end-state as mutually terminated, host-terminated, or parasite-terminated. If both host and parasite exhibited conditions for termination (condition 1 or 2) within 5 seconds of the other, an interaction was considered “mutually terminated.” At time-scales greater than this, it was clear which actor terminated the interaction due to ongoing conflict behavior by the other actor.

We stress, however, that the conditions to terminate for a host are different than for the parasite because there is only one parasite but five host ants. Any given host must meet the criteria relative to the single parasite, while the parasite must meet the same criteria, but relative to all host ants in their vicinity. If individual hosts met the above criteria, and this did not result in the complete cessation of an ongoing interaction which included other host ants, these were considered as “exits” rather than “interaction termination,” because hosts could rejoin an ongoing interaction at a later point.

### Analysis of host-parasite interactions: Behavioral summary, interaction initiation and escalation, parasite venom-use strategy, host response, and interaction termination

**Statistical framework and rationale.** To account for replication of colonies between pairings, a generalized linear mixed modeling (GzLMM) approach was used (*lme4, ordinal*; [37,38]). We included the effects of focal (host) colony as well as the “insert-ant” (parasite) colony as random effects in all GzLMM analyses. The effect of interaction (video) was also included in several GzLMMs where noted. For these analyses, the package *‘car’* was used to test for statistical significance of the main effects [39]. Each behavioral analysis used the complete set of 51 videos as a basis unless otherwise noted where the test statistic is reported in the results section. All analyses were carried out in *R*, using *Rstudio* [40,41].

**Comparison of direct and indirect venom use.** To understand whether parasites use direct and indirect venom with similar frequency, we assessed whether the count per interaction of direct stinging or indirect venom behaviors differed across interactions (i.e., all 1^st^ naïve interactions). To accomplish this, we used a generalized linear mixed model with parasite venom-use counts as the response variable and type of venom-use behavior (indirect or direct) as the predictor variable, and colony ID as a random effect. We used a Poisson distribution with a log link function.

**Sequence of associated conflict behaviors.** To identify conflict behaviors that may be serially associated, or which may act as potential stimuli for other behaviors within interactions, we assessed the order of the “first use” of each conflict behavior at the aggregate level. To accomplish this, we paired the first occurrence of each conflict behavior relative to the first occurrence of each other conflict behavior within a given interaction. To assess the relative order within each pairing, we tested whether each paired behavior occurred before or after each focal behavior using a GzLMM. For this, we used a binomial distribution with a logit-link function, including random effects for colony ID. Only unique behavioral pairings were included in calculating the corrected error rate; for instance, host biting relative to gaster-tuck sting and gaster-tuck sting relative to host biting are the same pairing, with the signs reversed, and so do not represent two different tests. We included host-host, host-parasite, and parasite-parasite behavior pairings in this analysis. Because separate tests were performed for 21 pairings, we used a holm correction to account for the family-wise error rate.

**Relationship between venom use and host behavior.** To understand if there is a relationship between host aggression and parasite venom use, we performed a GzLMM analysis (see Supplement S2 Appendix for methods, results). To understand the relationship between parasite venom use and host submission, we also performed a GzLMM analysis (see Supplement S2 Appendix for methods, results).

**Escalation of aggression between host and parasite.** We considered whether hosts or parasites escalated encounters into aggressive interactions, using several metrics. We specifically evaluated use of direct, offensive behaviors (starred, Table 1), including stinging (i.e., host: charge/lunge, bite, pull, carry; parasite: bite, side-swipe sting, gaster-tuck sting). We expected that, if one species were a clear aggressor, it would use these behaviors more frequently within interactions and use them in more interactions. We performed three analyses of these data: (1) We tested whether the frequency of direct offensive behaviors differed between species using a GzLMM with a Poisson distribution and log-link, with direct-offensive behavior counts as the response variable and actor as the predictor variable. (2) We tested whether hosts and parasite differed in whether they used at least one direct conflict behavior (i.e., engaged directly) using a GzLMM with a binomial distribution and logit-link. Actor type (i.e., species) was included as a predictor and host colony ID, parasite colony ID and video ID as random effects. (3) We also assessed whether host or parasites escalated to direct-offensive behavior.

**Parasite behavior following host biting.** To establish the effect of host biting on parasite venom-use behaviors (i.e., raised gaster, gaster flagging, side-swipe sting, gaster-tuck sting), we assessed venom behavior counts between interactions. To analyze parasite response when biting was absent versus present, we used a GzLMM with parasite venom-use counts as the response variable and host bite (absent or present) as the predictor variable to test mean venom use per interaction (across all interactions) for each venom behavior. For this, we used a Poisson distribution with a log-link function. Because we tested the effect on four dependent variables, we also performed a holm correction for the family-wise error rate.

**Parasite venom use relative to conflict escalation.** To assess whether parasite venom use changes in response to conflict escalation, we assessed venom-use behaviors across interactions with respect to host aggression. We aggregated occurrence of raised gaster, gaster flagging, side-swipe sting, and gaster-tuck sting that occurred before or after the first bite in each interaction. If no bite occurred in an interaction, parasite venom-use behaviors were coded as occurring prior to the first bite. Then, to test for a difference in parasite venom-use strategy between bite conditions, we performed an ordinal (cumulative-link) mixed regression analysis where the response variable was indicated as highest-ranked venom-use category occurring within an interaction, and the predictor variable was represented by bite position (before or after) relative to the highest-ranked response observed. For this analysis we used a logit-link function. Venom-use behaviors were ranked in terms of escalation based on evaluation of reciprocal behavioral pairings. We report the log odds change per escalation category and present the prediction and confidence value for before/after bite.

**Host submission following venom use.** To establish whether parasite venom use influences host behavior during engagements, we compared host submission counts between interactions with and without venom-use behavior. Because venom-use behavior can co-occur within interactions, we used a GzLMM with host submission counts as the response variable and presence/absence of each parasite venom-use behavior as the predictor variable. For this, we used a Poisson distribution with a log-link function. Since the amount of data for certain submission behaviors within venom-use categories was small, we combined data for individual host behaviors into the more general “submissive” grouping that included backing up, turning, and antennal tucking (Table 1). We report population marginal means and confidence intervals per condition [42,43].

**Interaction termination.** We also investigated whether venom use affects how interactions end. To do so, we examined 1) whether hosts or parasite terminated an interaction, 2) the escalation of venom use within the interaction (i.e., none, indirect, direct, see Table 1), 3) the presence/absence of host submission behavior, and 4) what host and parasite behavior directly precedes termination of the interaction. Termination occurred when an actor broke from interactions resulting in final cessation of conflict within a given interaction; although interactions can be comprised of multiple hosts (and temporary or non-terminal exits), we used only the final break for this analysis. With these data, we assessed two types of questions: 1) We tested whether hosts and parasites terminated a similar proportion of interactions overall (excluding mutual terminations) using a GzLMM with a binomial distribution and logit-link. Actor type (i.e., species) was included as a predictor and host colony ID, parasite colony ID and video ID as random effects, to test whether the intercept of the model differs from zero. 2) Using presence/absence data, we tested whether the proportion of clear host vs parasite terminated interactions were similar given any occurrence of host bite, parasite venom-use, or host submissive behavior. For this, we used a GzLMM with a binomial distribution and logit-link. Presence/absence of the focal behavior was included as a predictor and host colony ID, parasite colony ID and video ID as random effects. We excluded mutual terminations from hypothesis testing because there is ambiguity regarding if these terminations are truly mutual, or independently initiated at small timescales.

### Venom limitation

To characterize the costs of parasite venom use within the context of interactions, we assessed limitations to parasite venom usage in terms of 1) parasite anatomical venom storage, and 2) host neutralization capacity. We assume that parasite venom supply is not immediately replenishable, that the rate of production is slow relative to the timescale over which host-parasite interactions occur, and that host mortality is not immediate.

To establish venom storage capacity (in “number of stings”) we calculated the relative ratio in size between the individual droplets discharged by parasites directly from their sting and the total venom liquid stored in the venom sac (Supplement S3 Appendix). To assess the number of hosts a single parasite can neutralize using venom directly (stinging) we performed a combination of mortality assays using both whole venom sting and synthetic venom proxy (stereoisomeric mix of 3-butyl-5-hexylpyrrolizidine). We used the synthetic venom proxy to calibrate a lethal dose-response curve for hosts, which we used as a baseline model against which to compare mortality from whole venom, single stings. All analyses were carried out in *R*, using *Rstudio* [40,41]. Note, that ‘sting’, in this analysis, refers to the droplet size that is produced by parasites under anesthetized conditions; however, parasite individuals may have finer control over their venom delivery when using venom under normal conditions [31].

**Venom storage.** To calculate parasite venom capacity, as number of stings, we used venom droplet and venom sac content measurements (Supplement S3 Appendix) to obtain a ratio of venom sac to venom droplet size. To do this, we measured the area of the venom liquid dispersed on a 2-dimensional plane, where the exact height of the volume is not considered. It is important to note that this produces a “relative size” value. We also report the mean and 95% confidence interval for this sting capacity ratio per colony. To obtain the mean sting capacity, we calculated the ratio of mean venom sac size and mean droplet size per colony. We also computed confidence interval upper and lower bounds using a confidence level value of 1.96 and a sample size equal to the smallest count recorded between the venom droplet and venom sac measurements per colony. We used the per-colony sample means to calculate an overall species mean for parasite venom-storage capacity.

To obtain a measurement of parasite sting content, we dispersed the volume from individual venom droplets onto a 2-dimensional silica surface where we were able to measure the dispersion area of the 3-dimensional venom droplet with a standardized height. For these measurements we sampled between five and 24 *M. symmetochus* workers from four colonies (n_total_ = 41; Supplement S1 Appendix). Following methods described in [44], *M. symmetochus* parasites were first anesthetized over ice. Once anesthetized, a single drop of venom was everted from the parasite using a microcapillary tube. Each venom droplet was then applied to TLC silica gel 60 F254 strips stained with iodoplatinate reagent (*MiliporeSigma*, Burlington, MA). Once applied, strips were imaged, and the area of each droplet was calculated using *ImageJ* software [45].

To obtain a comparable measurement of parasite venom sac contents (S3 Fig), the full volume of venom from dissected venom sacs was dispersed onto 2-dimensional silica surface. For these measurements we sampled between three and eight *M. symmetochus* workers from the same four colonies (n_total_ = 22; Supplement S1 Appendix). Before dissection, ants were anesthetized, and TLC silica gel strips prepared in the same manner as they were for measuring droplet amounts. Ants were dissected by grabbing the sting with forceps and swiftly pulling. Ants were then dropped in chilled 98% ethanol and the venom apparatus was placed on the silica strip. Venom sacs were then punctured. Once applied, silica strips were imaged, and the area of each venom sac was calculated using *ImageJ* software [45].

**Toxicity of whole-venom stings.** *M. symmetochus* have spatulate stingers which are unable to penetrate their host ants’ exoskeleton. During conflict grappling, hosts may be stung anywhere on their body, and preliminary data suggest that whole venom stings to either the head or gaster resulted in similar levels of host mortality. Therefore, during mortality assays venom was applied to each host externally, to the dorsum of the host gaster.

To assess the toxicity of *M. symmetochus* whole-venom stings, using whole venom, we performed direct sting assays on *S. amabilis* host ants following methods similar to those in [44]. For this analysis, we use hosts and parasites from a colony found parasitized *in situ* (VAS190516−01). 30 Hosts and 20 parasite ants were selected, haphazardly, from the surface of the fungal garden and anesthetized over ice. Once anesthetized, a single drop of venom was everted from one of the 20 parasites and applied to each anesthetized host ant. Each *M. symmetochus* individual was used for a single application of a single venom droplet applied to one host ant. These parasites were returned to their colony following a 10-minute waiting period. Ten host ants were reserved for a mortality control, to which no venom droplet was applied but were each manipulated in the same manner, but with an empty microcapillary tube.

To determine mortality, after venom application, host ants in the same treatment and colony were placed together (with venom applied or without venom applied) inside 5 cm Petri dishes fitted with a piece of damp filter paper to retain humidity and prevent death via desiccation. Initial reactions were observed immediately following treatment, and again at 4 and 24 hours. Plates were tipped to the side during each check; mobile or standing individuals were recorded as “alive”, and overturned individuals were recorded as “alive/incapacitated” if further direct agitation produced a response (e.g., leg or antennal movement), or “dead” if unresponsive. Only the 24-hour mortality data was retained, since mortality continued to develop beyond 4 hours and the control group remained alive during the full observation period. These methods were applied for both whole venom and synthetic venom assays (described below).

**Toxicity of synthetic venom.** The active component of *M. symmetochus* venom, is a stereoisomeric mix of 3-butyl-5-hexylpyrrolizidine [26]. To perform lethal-dose assays for dose-response calibration, 3-butyl-5-hexylpyrrolizidine was synthesized by Tappey Jones (Virginia Military Institute) following the methods described in Jones *et al*., 1991 [46]. Initially, 5,8,11-heptadecatrione was prepared by the Stetter reaction of 4-oxooctanal and 1-nonen-3-one in the presence of a thiazolium salt catalyst. The triketone was purified by chromatography and kugelrohr distillation and subjected to reductive amination with sodium cyanoborohydride in the presence of ammonium acetate to provide 3-butyl-5-hexylpyrrolizidine.

A stock solution of 3-butyl-5-hexylpyrrolizidine was created by diluting 433 mg of the pure alkaloid into 5000 ml 96–98% molecular grade ethanol (*Fisher Scientific*), resulting in a final concentration of 86.6 μg/μl. Additional dilutions were then made from this chemical stock to obtain a set of solutions representing the full range of host mortality based on preliminary trials 3.2 μg/μl to 17.8 μg/μl (3.2 μg/μl, 5.6 μg/μl, 10.0 μg/μl, 13.4 μg/μl, 17.8 μg/μl).

To calibrate a dose-response curve for the active component of *M. symmetochus* venom, we performed lethal-dose assays on *S. amabilis* host ants using synthetic venom dilutions outlined above. An LD value approximates the dose required to induce mortality in X% of test subjects and is a standard measure to quantify acute toxicity of a substance.

For each dilution prepared (above) and an ethanol control, in most cases 10 host ants per treatment level, per colony were administered 0.5 μl of test solution (n_total_ = 339; Supplement S1 Appendix). For colony, RMMA190506−01, 6 ants were used per treatment, and one treatment contained 5 individuals (17.8 μg/μl), because there were too few individuals to assay 10 ants per treatment (Supplement S1 Appendix). When administering the treatment, 0.5 μl of liquid was applied to the external dorsum of the gaster using a 0.5 μl microcapillary tube. After the test solution was administered, subjects were placed together into Petri dishes, by treatment, where mortality was determined 24 hours after application (as above in the whole venom protocol). No mortality was observed in the control groups during this experiment.

Mortality resulting from synthetic alkaloid exposure was modeled in *R* using the *drc* package (Supplement S4 Appendix; [47,48]). A lethal dose value is calculated by solving the dose-response formula for the value of *x,* given a mortality value *f(x)* between 0 and 100%. To approximate the upper limit on host 3-butyl-5-hexylpyrrolizidine alkaloid tolerance, we calculated the LD_90_ value directly from the dose-response curve. Finally, we then estimated the dose, *x,* of active 3-butyl-5-hexylpyrrolizidine present per *M. symmetochus* whole-venom sting using the same formula with the whole venom mortality rate *f(x)* obtained from the single sting assays.

We determined host neutralization capacity by calculating the relative difference in effective dose between a single whole-venom sting, and that required to achieve 90% host mortality. We then used these results as a scaling factor to convert total venom storage, in “number of stings,” to host neutralization capacity in “number of hosts.” Because our venom storage calculations are based on a distribution of measurements, we present these data as a mean with estimated 95% confidence intervals.

## Results

### Venom use is biased toward indirect venom behaviors which occur prior to stinging

**Fig 1:**
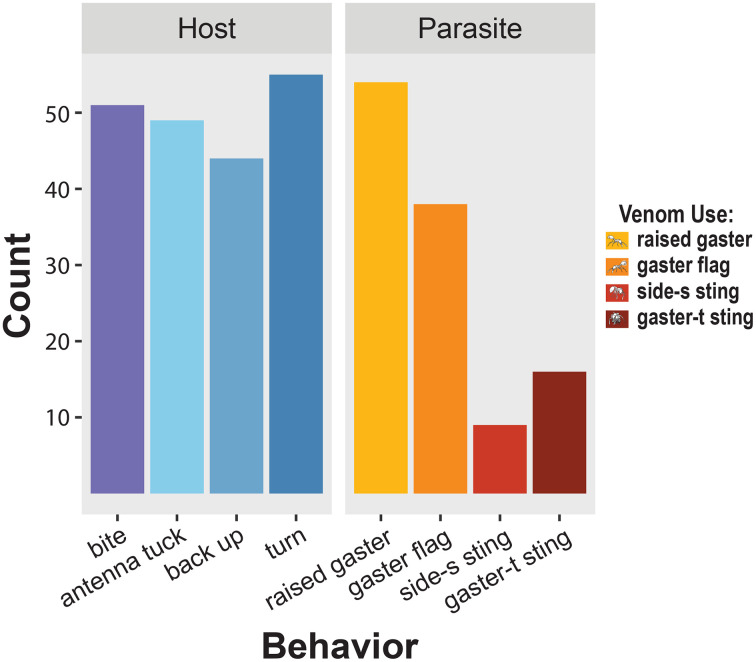
Sum of venom-related behavior across all interactions. The set of interactions investigated includes only the first, naïve interactions to occur in each arena trial. Host behavior studied ranged from aggressive (bite) to submissive (antenna-tuck, back up, turn). Parasite behavior studied involves indirect alkaloidal venom use (raised-gaster, gaster flagging) and direct stinging behavior (side-swipe sting, gaster-tuck sting). See Table 1 for full list of behaviors/descriptions.

When investigating host and parasite conflict behavior, we found that host biting and submissive behaviors (i.e., antenna tuck, back up, turn) occurred in similar proportions to indirect venom-use behaviors (i.e., raised gaster, gaster flagging), which all occurred more frequently than parasite stinging. Overall, parasites used indirect venom behaviors 3.68 times more than direct stinging behaviors (n_(indirect)_ = 92, n_(direct)_ = 25; Fig 1). We found a significantly greater mean indirect venom count across interactions (GzLMM, venom type: N = 51, Wald χ^2^ = 35.159, DF = 1, p_(main effect)_ < 0.001).

**Fig 2:**
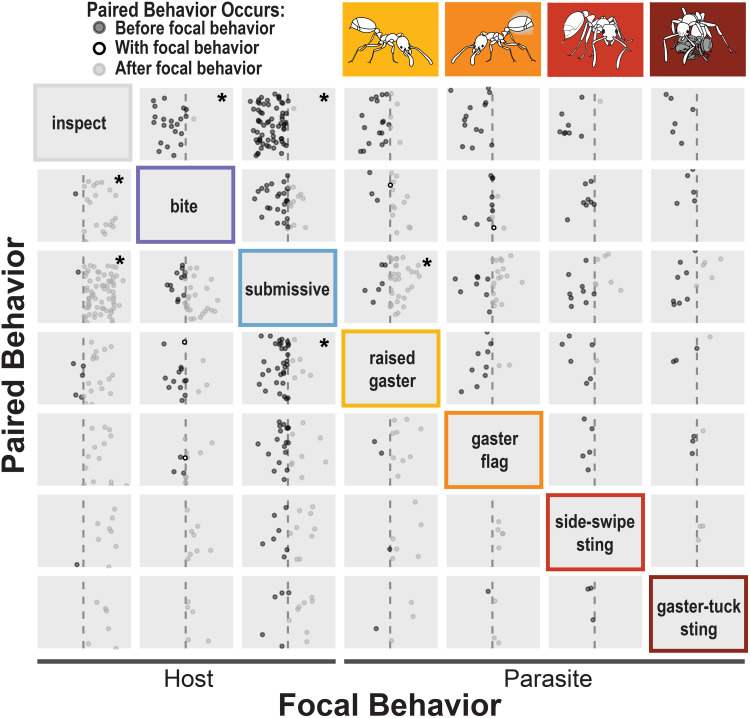
Pairwise plots of time differences between the first occurrence of each behavior. Each dot shows the timing (before vs after) of the first occurrence of a behavior if it co-occurred with the focal behavior within an interaction. Each panel aggregates all (first) co-occurrences of two behaviors across all interactions and demonstrates the relative timing (proportion before vs after) of the paired behavior relative to the focal behavior. Column refers to the focal behavior and row refers to the paired behavior. Dashed mid-lines reference the occurrence of the focal behavior in a given interaction. To aid in visualization, time differences on the x-axis between behaviors are z-score normalized to +/-3 standard deviations. Panels are marked with their significance level according to GzLMM testing, at alpha = 0.05 using a Holm correction for multiple comparisons. Significance, marked with an asterisk, is shown on both sides of the diagonal as they represent reciprocal pairings. Each column can be viewed as a group to observe the relative timing of each paired behavior to the focal behavior along the dashed-vertical line. Each row can be read to compare the relative timing of that behavior (proportion grey vs. dark grey) against other behaviors indicated by their central dash-lines. Submissive-type behaviors are grouped into the category “submissive.” Host/parasite behaviors follow the same color scheme as Fig 1; host behaviors are cool, while parasite venom behaviors are warm.

We paired the first observations of each host and parasite conflict behavior whenever they co-occurred in interactions. The “first-use” of each behavior, relative to the first-use of every other behavior that co-occurred in that interaction, is shown on Fig 2. The relative timing of parasite venom behaviors to host behaviors can be seen on the lower left, or upper right quadrants. We found that the first-use of parasite raised-gaster behavior was significantly more likely to occur before host submission behaviors. However, the proportion of venom behaviors observed before each host behavior declined between raised-gaster and gaster-flagging behavior, and still further versus direct stinging behaviors (Fig 2, columns 1–3, lower half). Following this same trend, the indirect venom-use behaviors (i.e., raised gaster, gaster flagging) generally occurred prior to direct stinging (Fig 2, lower right quadrant).

The relative timing of host behaviors to parasite behaviors can be seen on Fig 2. We found that the first-use of host inspection occurred significantly earlier than host bite, host submission, and also significantly earlier than parasite gaster flagging. Host inspection behavior preceded all other conflict-related behaviors most of the time (Fig 2, column 1). In addition, we found that hosts always bit before parasites stung (Fig 2, column 2, panels 6 & 7). Finally, comparing between panels (Fig 2, row 3, panes 4 & 5 vs 6 & 7), although the first host submissive behavior generally occurred after the first parasite venom usage—the first submission often took place before any stinging when both were observed, and the number of interactions in which parasite stinging co-occurred did not account for the number of interactions across which host submissive behavior was observed. Instead, the majority of first submissive behaviors appeared after first-use of indirect venom (Fig 2, row 3, panes 4 & 5 vs 6 & 7).

### Heterospecific aggression is initiated by host ants and influences parasite behavior

We also evaluated how frequently direct-harm behaviors were used in conflict and during conflict escalation. In general, we found a positive relationship between host aggression and parasite venom-use behavior (Supplement S2 Appendix, S2 Fig, panel a). We found that host ants were the primary aggressor in heterospecific interactions. Overall, hosts, despite lacking a sting, exhibited 3.56 times more direct, offensive behaviors (i.e., host: biting, charging, lunging; parasite: stinging) than parasites (n_(host)_ = 89, n_(parasite)_ = 25). There was a significant effect of actor type on the frequency of direct, offensive behaviors (GzLMM, actor type: N = 51, Wald χ^2^ = 27.506, DF = 1, p_(main effect)_ < 0.001). Similarly, we found that hosts were significantly more likely to engage directly in interactions (GzLMM, actor type: N = 51, Wald χ^2^ = 7.5344, DF = 1, p_(main effect)_ = 0.006). Hosts engaged in direct-offensive behaviors in twice the number of interactions than parasites did (n_(host)_ = 27, n_(parasite)_ = 13). Moreover, hosts were responsible for escalating encounters into direct conflict in 27 out of the 28 interactions where direct-offensive host behaviors occurred.

**Fig 3:**
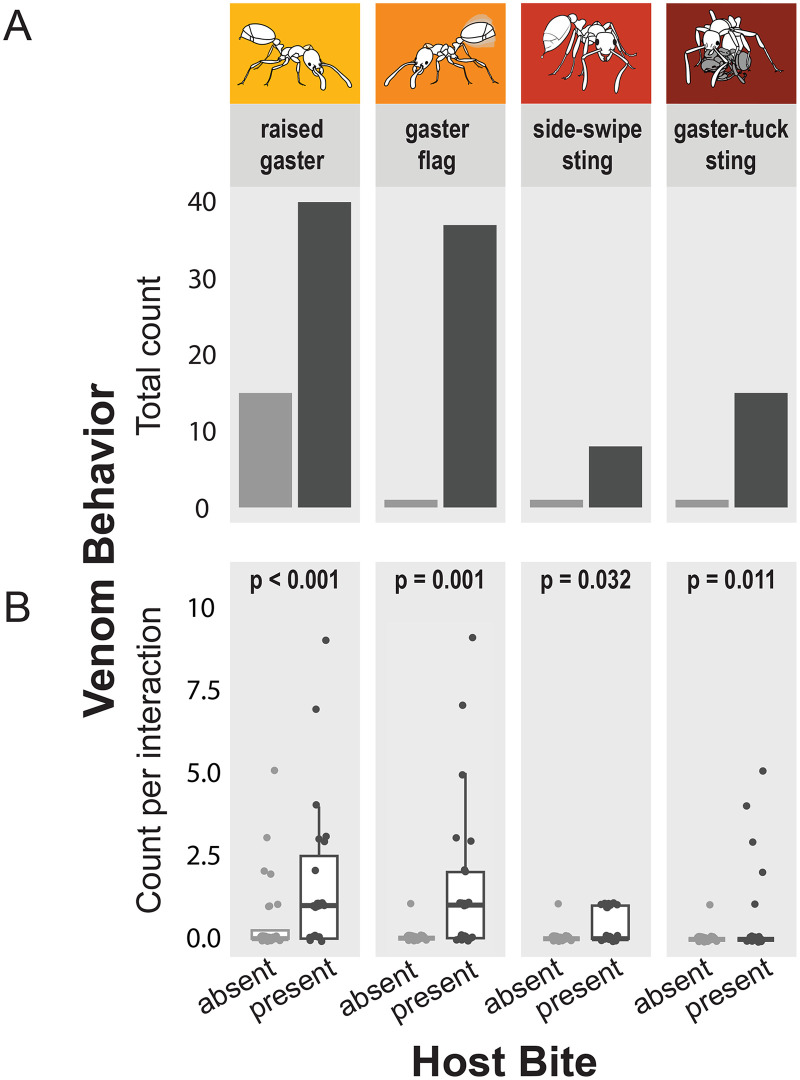
Co-occurrence of host biting and parasite venom behaviors. Data for videos in which host bite was absent (n = 23) is presented in light grey, while data for videos in which host bite was present (n = 28) is shown in dark grey (N_(total)_=51 videos). **A)** Aggregated total of parasite venom usage across interactions where host bite was either absent or present from an interaction. Differences in both total count and relative proportions between bite conditions are visible between venom-use behaviors**. B)** Difference in mean parasite venom usage per interaction when host biting was absent versus present was tested using GzLMM. Four models were fit (one per venom-use category) and significance was assessed using a holm correction for multiple tests.

Given the above, we still found that parasites used all venom behaviors significantly more frequently when host biting was present in an interaction (Fig 3a, b). However, we also discovered that an indirect venom behavior, raised gaster, was the only venom-use behavior used appreciably in interactions where biting did not occur (Fig 3a, column 1 vs. 2–4).

### Parasite venom-use shifts in response to host escalation

**Fig 4:**
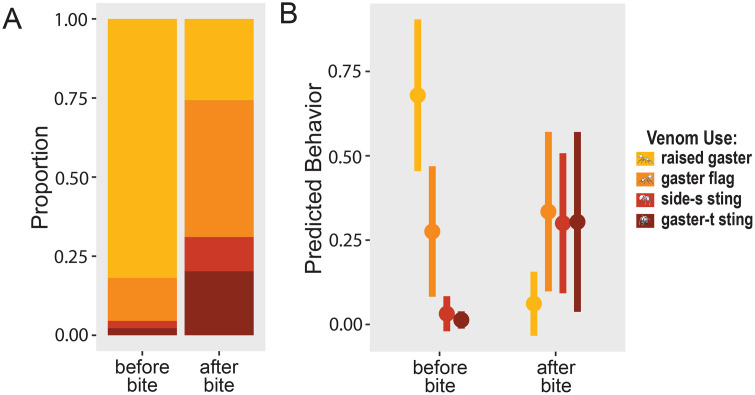
Parasite venom-use response. **A)** Change in venom-use behavior following the first host bite within an interaction. Profiles are compiled from parasite venom-use behavior data (raised gaster, gaster flagging, side-swipe sting, gaster-tuck sting) aggregated across all interactions and binned based on whether host bite has yet occurred within a particular interaction. The category of “other” is excluded (see Table 1). **B)** GzLMM predicted venom behavior before vs after host bite, shown with 95% confidence intervals. Venom-use behaviors were ranked in order of escalation using data presented in 4a and in Fig 2 (raised-gaster < gaster flag < side-swipe sting < gaster-tuck sting) and modeled using a cumulative-link mixed model.

To characterize how parasites strategically use venom with respect to host escalation, we compared the frequency of parasite venom-use behaviors before and after the first occurrence of a host bite. We found that the parasite venom-use profile changed after biting occurred (Fig 4a). Although parasites used indirect venom behaviors before and after bite occurs, raised gaster was the predominant behavior observed before hosts escalated, while gaster flagging was predominant after host aggression. Direct venom behaviors (i.e., stinging) were rare prior to host escalation, and was only modestly elevated even after escalation (Fig 4a). We found that occurrence of biting significantly increased the chances of observing escalated venom-use behavior by parasites (GzLMM, bite: N = 38, Wald χ^2^ = 21.853, DF = 1, p_(main effect)_ < 0.001). Specifically, the odds of observing a higher-ranked venom-use behavior become >30 times greater once host bite has occurred. The predicted probability of observing a specific venom behavior before or after bite is shown in Fig 4b. Raised-gaster is predicted before bite, while gaster flagging or stinging is predicted to occur after bite. Odds ratios for ranked venom behaviors are provided in Table 2.

**Table 2:** GzLMM odds ratios of cumulative link thresholds for ranked venom behaviors, occurring after bite.

		Ordinal factors	
Predictors	Odds Ratios	CI	p
after bite	32.38	5.56 - 188.62	**<0.001**
raised-gaster | gaster-flag	2.12	0.75 - 5.95	0.154
gaster-flag | ss sting	21.22	3.87 - 116.26	**<0.001**
ss sting | gt sting	74.1	10.58 - 519.06	**<0.001**

The overall odds of observing a higher ranked venom behavior for each increase in level of the predictor variable is provided before the individual thresholds. Threshold odds, listed second, are based on the likelihood of meeting a given threshold or below. Significance is denoted by bold type.

### Venom alters host behavior

**Fig 5:**
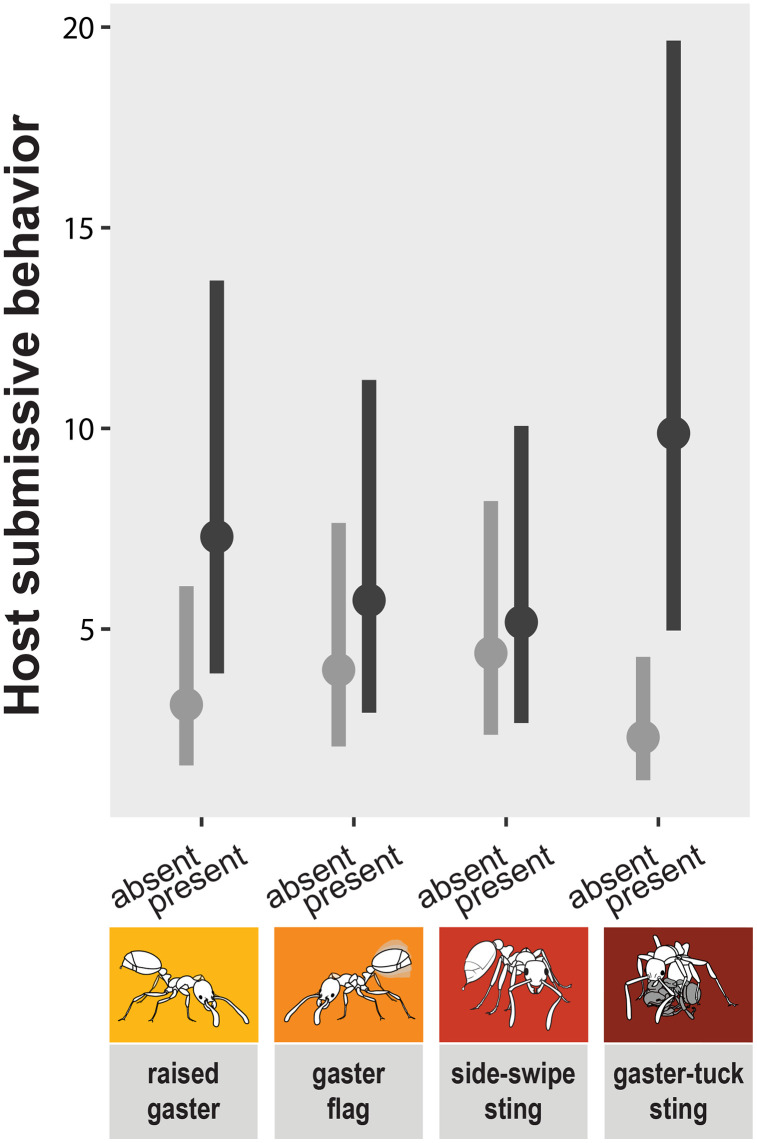
Effect of parasite venom behavior on host submission. Estimated host submission events per interaction when parasite venom behaviors are absent (light grey), versus present (dark grey), in an interaction. Model estimated marginal means are indicated by a dot with 95% confidence intervals.

We evaluated host submission response to parasite venom use across each venom behavior. Overall, we found that both direct and indirect parasite venom-use behaviors are strongly and positively correlated with host submission rates (Supplement S2 Appendix, S2 Fig, panel b). We also found that host submission counts were significantly greater when raised gaster or gaster-tuck sting were present within interactions than when these were absent. However, we did not find a significant effect of the presence of gaster flagging or side-swipe stinging on host submission (Fig 5, Table 3).

**Table 3:** ANOVA (Analysis of deviance) results for a GzLMM of parasite venom effect on host submission.

Parasite behavior	LR Chisq	df	Pr(>Chisq)
raised-gaster	15.08	1	**0.001**
gaster-flag	1.89	1	0.169
side-swipe sting	0.60	1	0.435
gaster-tuck sting	34.19	1	**0.001**

Test values for differences in mean host submission count per interaction with or without venom behavior presence. Significance is denoted by bold type.

### Interaction termination is more often initiated by parasites after venom-use behavior

We assessed termination by either species in the context of prior conflict behavior (Supplement S5 Appendix, S5 Fig). Overall, we found that termination by parasites was more frequent than termination by hosts (38 vs. 22 interactions). Excluding mutual termination, parasites ended interactions 2.78 times more often than hosts, overall (n_(host)_ = 9, n_(parasite)_ = 25, S5 Fig), and were significantly more likely to terminate than hosts (N = 34*,* p_(main effect)_ = 0.0275)p < .01).

We also assessed termination, per species, based on the presence/absence of specific conflict behaviors (host bite, parasite venom, host submission). We did not find a significant effect of host biting on whether hosts or parasites terminated interactions (N = 34*,* p_(main effect)_ = 0.5858; S5 Fig, layer 1). Although we did not find a significant effect of venom use on whether hosts or parasites terminated (N = 34*,* p_(main effect)_ = 0.0669), this p-value approached our alpha threshold (0.05), and venom use was much more common when parasites terminated (21 out of 38 interactions, vs. 15 out of 25 excluding mutual termination) than when hosts terminated (6 out of 22 interactions, vs. 2 out of 9 excluding mutual termination; S5 Fig, layer 2).

While parasite venom-use behavior frequently co-occurred with host submission, it also frequently co-occurred with parasite termination (Supplement S5 Appendix). However, we did not find a significant effect of host submission on actor termination (N = 34, p(main effect) = 0.796; S5 Fig, Layers 3–5). Submissive host behaviors were present regardless of who terminated interactions and, in some cases, occurred independent from either host aggression, or parasite venom behaviors (S5 Fig).

### Parasites can lethally envenomate a small number of hosts

To explore whether venom can be costly for parasites to use during interactions with their hosts, we evaluated 1) parasite anatomical venom storage capacity, and 2) host neutralization capacity (in “stings”). By combining whole-venom sting and toxicity assays, we quantified parasite storage capacity in “stings” and translated these measurements into an estimate of the number of hosts a parasite can lethally envenomate via direct stinging.

**Fig 6:**
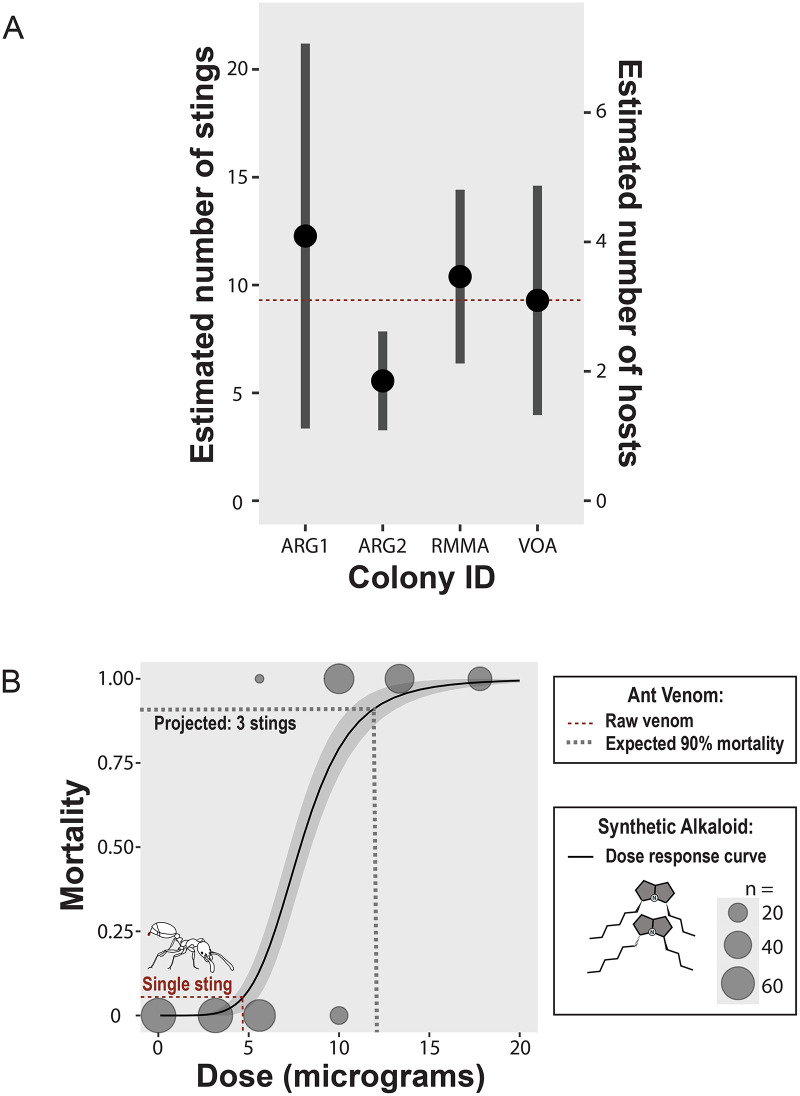
Parasite venom storage and host neutralization capacity. Parasites are estimated to be able to neutralize three hosts under laboratory nest conditions. **A)** Parasite venom storage (in stings) and lethal envenomation capacity (number of hosts). Estimated mean and 95% confidence interval for relative size (magnitude difference) between single whole venom stings and dissected venom sacs (Supplement S3 Appendix, S3 Fig) for four colonies of *M. symmetochus* parasites. The overall species mean is presented as a red, dotted line. The right-hand scale converts raw sting number by a factor of three based on the venom cost per host (shown in 7b). **B)** Venom cost (in stings) per host. A dose-response curve was projected based on a chemically synthesized 3-hexyl-5-butyl-pyrrolizidine alkaloidal venom proxy (LD_90_ = 11.65 μg, n_colonies_ = 7, n_total_ = 339). Mortality count resulting from venom proxy at each dose is represented by circle size along the top and bottom of the figure (mortality was scored as binary 0 or 1, alive or dead, respectively). Mortality rates from whole venom, single-sting assays (red, dashed) indicate that parasites use approximately 4.62 μg active alkaloid per sting. At this dose, it would require at least three stings to achieve 90% probability of host mortality (grey, dotted) using the projected lethal dose model.

Parasites were estimated to carry 9.3 droplets of venom, on average, within their venom sac (Fig 6a, left axis; also see Supplement S3 Appendix). Mortality rates from whole venom, single-sting assays indicated that parasites use approximately 4.62 μg active alkaloid per sting. At this dose, it would require at least three stings to achieve 90% probability of host mortality using the projected lethal dose model (Fig 6b). This LD_90_ value converts to a lethal capacity of approximately three hosts per parasite, Fig 6a, right axis).

## Discussion

Here, we present evidence that *M. symmetochus* guest-ant social parasites use venom as a signal to their *S. amabilis* hosts. Because interspecific conflict in this system is costly and potentially lethal for both parties, venom signaling may reduce social costs associated with cohabitation for both colonies of a host-parasite pair. We suggest that signaling is favored by natural selection due to fitness interdependence resulting from long-term cohabitation and a common interest between parasites and their hosts in the face of more extreme exploitation by a third party [26].

We have shown that both indirect and direct venom-use behavior significantly alters host behavior during conflict interactions. However, *M. symmetochus* do not rely on stinging as a primary, offensive strategy. In general, parasites avoid stinging except as a response to host aggression. Although conflict is typically initiated by hosts and parasite venom-use shifts in response to host aggression, parasites predominantly use indirect venom behavior, both before and after host aggression. Raised-gaster behavior, specifically, is both commonly observed early in interactions before biting occurs and is the only venom behavior used appreciably in interactions where biting does not occur. Thus, we suggest that parasite raised-gaster behavior is a pre-emptive signal to hosts in response to potential risk, while the other parasite venom behaviors investigated (raised gaster, side-swipe sting, gaster-tuck sting) primarily function as responses to host aggression.

Although submissive host behaviors often preceded the termination of interactions by either species, parasites themselves often end interactions after venom use. We therefore conclude that parasites use venom, directly or indirectly, to elicit host submission—or else, to open a window of opportunity to exit an altercation when hosts do not exhibit submissive behaviors. While stinging is expected to enhance parasite survival if used lethally, when interactions are terminated in a way precluding full conflict escalation--either to host aggression or to parasite stinging--this should effectively reduce mortality risk for both hosts and parasites. The number, or ratio, of hosts actively participating in an interaction may be a critical factor influencing hosts’ decision to submit or escalate, though further study is necessary.

While we did not find evidence of increased host submission in response to gaster flagging and side-swipe sting presence, this may stem from specific conditions under which these behaviors are used in conflict. We suggest that gaster flagging is only used when hosts are “fully committed” to aggression, and therefore, could be predisposed against “backing down” at that juncture. The change in indirect venom behavior (additional flexing behavior observed) by parasites may include stridulating to volatilize or spread venom more rapidly using vibratory action, or possibly, as a call for help [49,50]. Side-swipe sting is often used in fringe attack cases—e.g., when the host has mounted an unsuccessful or poorly aligned attack—leaving an opportunity for the parasite to leave the vicinity (terminating the interaction) before the host might respond with submission. Therefore, further analysis of post-interaction behavior may further clarify these results.

Venom aerosolization has been observed in other ant species during gaster-flagging behaviors [31] and may constitute a threat of a parasite’s intent to defend itself. While *M. symmetochus* venom is toxic to hosts, we do not expect venom aerosolization behaviors—which volatilize from a single drop of venom [31]—to present lethally toxic levels of venom to hosts because they can terminate interactions by ceasing aggression at any time. However, nonlethal displays prior to conflict escalation are known to provide valuable information for combatants to assess risks of engaging and avoid conflicts they are unlikely to win [51,52]. Further, co-option of weaponry or other abbreviated forms of aggression is common in animal aggression signaling [53], and hymenopteran venoms can contain chemical components (e.g., volatiles, pheromones; [33]) that are known to transmit information in social insects [14,54].

Although gaster-flagging may present a credible threat of envenomation, we also found that the number of stings which *M. symmetochus* can deliver at once is limited, suggesting that parasites risk venom depletion if venom were used indiscriminately. Previous literature has also shown that *M. symmetochus* are outnumbered by *S. amabilis* by a ratio of 2:1 within nests [36], so parasites are likely to encounter multiple hosts simultaneously or sequentially within the nest. As a result, the modest host neutralization capacity of *M. symmetochus* individuals suggests that venom is an extremely valuable resource in terms of opportunity costs. This premium on parasite venom supply could help maintain signal honesty ([55]; but see [56–58]).

Our estimate of host neutralization capacity has several caveats; however, we expect that our values are conservative, and thus represent minimum costs that parasites pay during conflict. First, our estimates of parasite storage represents the average level of venom stored under average nest conditions, which may not reflect total physiological venom capacity of a single individual. Since *M. symmetochus* do not nest independently of their hosts and may also use venom to contribute to various nest functions [59], parasites may not hold maximum venom reserves, especially if conflict is occurring in the nest at a broader scale or under other conditions where venom may be used more frequently than normal. Second, our estimates of a parasite’s host-neutralization capacity via direct stinging may over-estimate host mortality on the time scales of an interaction. Mortality rates, as modeled, take hours to mature in the lab. As a result, additional venom would likely be required to neutralize hosts immediately, which would lower the expected neutralization capacity of parasites as calculated. *Sericomyrmex amabilis* hosts can remain active/aggressive for hours even after being dosed lethally, and individuals have been observed to recover post sting (MRB, *personal observation*), as in several other *Megalomyrmex* host species [18,19,26]. This would be expected to exacerbate venom costs to neutralize aggressive hosts, regardless of parasite’s estimated storage reserve.

Why then, do *M. symmetochus* use venom as a conspicuous, and seemingly honest, signal despite the apparent costs to individuals? At the colony level, signaling should reinforce the advantages of prudent exploitation by parasites. If *M. symmetochus* colonies do not compete with other colonies of the same species within a host nest (i.e., multiple infection as in [25,60]) parasites might obtain a long-term return on investment from strategies that reduce burden on their host colony. As larger host colony size is expected to facilitate host resource acquisition [22,23], parasites may be able to reap higher reproductive output in the future by reducing mortality on hosts at a given parasite load. Similarly, the risks of repeated, costly interactions could also incentivize venom signaling by parasite individuals. During conflict, host ants latch on to parasites with mandibles to mutilate or drag them—this impairs parasite mobility and increases probability of secondary attack by additional hosts. In these instances, parasites would be forced to neutralize multiple hosts if an interaction escalates. Since venoms/toxins are metabolically costly for organisms to produce [61], however, animals across taxa limit venom delivered to neutralize prey [62,63]. In species related to *M. symmetochus*, there is evidence that venom measured during gaster flagging can be much less than would be used for stinging [31], implying an ability to meter venom via this mode of delivery. As a result, in host-dominated social contexts, venom use could be reduced by precluding direct conflict while broadcasting communication to multiple hosts simultaneously while preserving costly venom against the risk of future encounters with hosts. Together, there could be strong incentive against stinging by parasites collectively, and consequently, selection for the evolution of a venom-based signal.

What are the advantages for *S. amabilis* hosts that attend or respond to venom as a signal? Dawkins & Krebs (1978) argue that misalignment of interests between signalers and receivers should favor signalers who manipulate (i.e., deceive) receivers, and there is a fundamental conflict over resource allocation between parasites and their hosts. However, individual hosts still stand to benefit from paying attention to venom signals by gathering information about the fighting ability of their opponent if the signal transmits reliable information [2] about parasite venom reserves (i.e., fighting ability), or affective state [56,64]. It has also been suggested that hosts may be able to recover fitness in the face of parasite exploitation when parasites use a “mafia-like” strategy (i.e., the parasite retaliates if the host resists; [65,66] but also see [67,68]). By reacting submissively, *S. amabilis* may lower (direct) mortality costs on the host colony when parasites do not resort to stinging. Although fitness gains must be traded off against the potential (indirect) costs associated with a higher parasite load, a reduction in host worker mortality could enhance host colony fitness in the long term (through worker resource acquisition, reproductive output, and environmental resilience; [22–24]). Finally, *M. symmetochus* guest ants will defend their host’s nest by using their venom directly (i.e., stinging) against raiding *Gnamptogenys hartmani* ants, which otherwise decimate host fungal food reserves [26]. As a result, *S. amabilis* hosts may be making “the best of a bad job” [69] by tolerating their parasite infection [70]. Since evicting the parasites outright may be too costly (or impossible) for hosts, living peaceably may be more advantageous to hosts than existing in a state of continuous, overt conflict; especially given the potential defensive benefit cohabitation provides to hosts.

As a result, both symbionts appear to be able to benefit from maintaining the association in a relatively low conflict state. Parasite reliance on host colony persistence, and the potential role that the parasites play in defending resources of the host colony, together, appear to result in a form of conditional interdependence. However, the level of benefit from signal use is not necessarily equally shared between a nest pair due to the different avenues by which each species benefits. In nature, the specific benefit of signaling to each species likely rests on the (intrinsic) social balance of power between hosts and parasites (e.g., as individuals versus in groups), in addition to the (extrinsic) local raiding pressure by *G. hartmani*. For example, high conflict impacts host resources and parasite load, but also parasite defensive potential (via the number of parasites and their venom storage). As a result, the propensity for parasite-host signaling should be correlated with raiding pressure but may also depend on features of the social structure between *M. symmetochus* parasites and their *S. amabilis* hosts.

It is unclear how often mutually-beneficial signaling might occur in other parasitic systems. Aside from the fitness interdependence present in this system (resulting from external predation), the *S. amabilis*/*M. symmetochus* symbiosis is also atypical in its internal social organization. As a result, the parasite signaling observed here may be quite exceptional. An abundance of organisms, however, are known to exploit social insect societies, and many nest associates (both unitary and social) have converged on strategies which “hack” social host communication networks in one way or another (e.g., eavesdropping, stealth, propaganda; [54,71]). In many ways, this broad strategy parallels more traditional parasitic systems, which exploit host signaling pathways to the symbionts’ reproductive advantage at the other molecular, cellular, or other physiological levels [72,73]. Yet, social parasite systems differ from other parasitic systems because they have intrinsic social components. For example, social parasites exhibit a variety of lifestyles with their hosts [13,14,74], and the social organization of the parasitism should influence both the costs of the parasitic association and any effects of signaling from within. Although it will be difficult to frame the diversity of parasitic systems with respect to their overlapping features, systematic investigation of signaling across parasite systems which vary in their scale, structure, and level of interdependence (see [75–79], may reveal unrealized examples of beneficial antagonistic signaling which could deepen our understanding of biological signaling more broadly.

To our knowledge, this is the first demonstration of a signal being used to coordinate interspecific social integration between a social parasite and their hosts, which highlights that communication strategies to manage conflict can arise naturally, even in antagonistic partnerships. While mutual reduction in costs can serve to align the interests of agents or groups with competing interests, the conditional nature of the benefit to each species in this system emphasizes the need for careful consideration of both intrinsic and extrinsic costs and incentives.

## Supporting information

S1 Appendix Table of colony codes and sample sizes for video and venom assays. (XLSX)

S2 Appendix Relationship between focal host and parasite behaviors. (DOCX)

S3 Appendix Relative difference in venom content between parasite venom droplet and dissected venom sac. (DOCX)

S4 Appendix Parameter estimates for a 4-parameter log-logistic lethal dose model based on host exposure to synthetic venom (3-butyl-5-hexylpyrrolizidine). (DOCX)

S5 Appendix Summary of interaction termination. (DOCX)
