# Supplementary material for: Guest-ant social parasites avoid conflict with social hosts using venom signaling
Source: PLoS One. 2026 Aug 12;21(8):e0345143. doi: 10.1371/journal.pone.0345143 (PMC13465834; doi:10.1371/journal.pone.0345143)
Supplement: Supplementary file 1 [file pone.0345143.s002.docx]

**S2 Appendix. Relationship between focal host and parasite behaviors.**

**S2 Text. Methods**

To establish whether nonrandom relationships exist between conflict behaviors, we assessed parasite venom use (stinging, gaster-flagging, raised gaster) and 1) host aggression (biting) or 2) host submission (sum of antenna-tuck, back-up, turn) behaviors using a mixed linear regression approach. We used a Poisson distribution with a log link function, including random effects for colony ID (Table S1). We used figure 2 as a basis to determine putative stimulus and response axes. For 1), we regressed the rate of host biting as the predictor variable, against the rate of parasite venom use as the response variable, for each venom behavior type (Fig S2, panel a, table S2). For 2), for each venom behavior type, we regressed the rate of parasite venom use as the predictor variable, against the rate of host submissive behavior as the response variable (Fig S2, panel b, table S2). For all regressions, we transformed behavioral occurrence data into a rate (occurrence per time duration of the interaction). For those regressions including parasite stinging behavior, we combined both parasite sting behaviors into a single “sting” category because few datapoints were observed for either direct venom-use behavior. For aggression-venom regressions, we include all interactions because host aggression is the putative stimulus for all venom behaviors. For venom-submission correlations, we included only interactions where only the focal venom behavior was used to isolate the effect of each venom behavior as a putative stimulus for submission behaviors.

**S2 Fig. Relationship between focal host and parasite behaviors.** Each panel corresponds to the host behavior specified at the top of the column, and the parasite venom behavior depicted on the right margin. The horizontal and vertical axes are based on the typical order of occurrence between the behaviors as a category (Figure 2). Each plot also depicts point density along each axis. **A)** Host aggression vs parasite venom behaviors. Regressions between rate of host biting (x axes) and rate of parasite venom use (y axes). Because parasites rarely use gaster-flagging unless a host bite connects fully, we analyzed the bite-gaster-flag correlation on only the subset of interactions where biting makes direct contact. Biting is positively and significantly correlated with each venom behavior. **B)** Parasite venom vs host submission behaviors. Regressions between rate of parasite venom-use (x axes) and rate of host submission (combined antenna-tuck, back-up, turn; y axes). Each venom-submission panel is subset by interactions where only that venom behavior is present. In all cases we find significant, positive relationships to host submissive behaviors.

B

A

**S2 Table. ANOVA results for GzLMM regression analysis of focal host and parasite behaviors.** Significance is denoted by bold (.05) and italic (.001) text.

| **GzLMM ANOVA** | |  |  |  |  |  |
| --- | --- | --- | --- | --- | --- | --- |
| **Host bite (rate)** | **Parasite venom (rate)** | | **Chisq** | **Df** | **Pr(>Chisq)** | **sigf** |
| host bite | parasite raised gaster | | 28.008 | 1 | ***<0.001*** | ********* |
| host bite | parasite gaster flag rate | | 47.984 | 1 | ***<0.001*** | ********* |
| host bite | parasite sting rate | | 10.729 | 1 | **0.001** | ******** |
|  |  | |  |  |  |  |
| **Parasite venom (rate)** | **Host submission (rate)** | | **Chisq** | **Df** | **Pr(>Chisq)** | **sigf** |
| raised gaster | host submission | | 12.224 | 1 | ***<0.001*** | ********* |
| gaster flag | host submission | | 22.789 | 1 | ***<0.001*** | ********* |
| sting | host submission | | 10.454 | 1 | **0.001** | ******** |
