# Supplementary material for: Guest-ant social parasites avoid conflict with social hosts using venom signaling
Source: PLoS One. 2026 Aug 12;21(8):e0345143. doi: 10.1371/journal.pone.0345143 (PMC13465834; doi:10.1371/journal.pone.0345143)
Supplement: Supplementary file 2 [file pone.0345143.s003.docx]

**S3 Appendix. Relative difference in venom content between parasite venom droplet and dissected venom sac.**

**S3 Fig. Relative difference in venom content between parasite venom droplet and dissected venom sac**. Venom droplets and dissected venom sacs were measured as the surface area of a 2-dimensional disc after transfer onto silica gel. Parasite venom capacity was calculated from the relative area where venom capacity = venom sac/venom sting size. Colony reference codes are shown above each panel.
