# Supplementary material for: Guest-ant social parasites avoid conflict with social hosts using venom signaling
Source: PLoS One. 2026 Aug 12;21(8):e0345143. doi: 10.1371/journal.pone.0345143 (PMC13465834; doi:10.1371/journal.pone.0345143)
Supplement: Supplementary file 3 [file pone.0345143.s004.docx]

**S4 Appendix. Parameter estimates for a 4-parameter log-logistic lethal dose model based on host exposure to synthetic venom (3-butyl-5-hexylpyrrolizidine).**

**S4 Text. Methods**

Synthetic alkaloid mortality data were modeled using a four-parameter log-logistic function with fixed lower and upper limits (0, 1).

$f\left( x \right)=c+\frac{d-c}{1+exp(b\left( \log\left( x \right)-\log\left( e \right) \right))}$	(EQ 1)

Where *b* represents the logistic growth rate (slope), *c* is the lower limit, *d* is the upper limit, and *e* represents the value of the sigmoid inflection point. A mixed-effect logistic model was used, including random effects for colony ID. Model fit was assessed using a lack-of-fit test based on Pearson’s statistic in the R package *drc* (Ritz *et al.* 2005, 2015). A lethal dose value is calculated by solving the dose-response formula for the value of *x,* given a mortality value *f(x)* between 0 and 100%.

**S4 Table. Parameter estimates for a 4-parameter log-logistic lethal dose model based on host exposure to synthetic venom (3-butyl-5-hexylpyrrolizidine).** Lower and upper parameters were set to zero (no mortality) and one (complete mortality), respectively. As this model reduces to a 2-parameter model, estimates are provided here for the logistic growth rate (slope, *b*), and the value of the sigmoid inflection point (*e*). Significance is denoted by bold (.05) and italic (.005) text.

| **Parameter** | **Estimate** | **Std. Error** | **t-value** | **p-value** |
| --- | --- | --- | --- | --- |
| b:(Intercept) | -5.56177 | 0.62086 | -8.9581 | ***0.001*** |
| e:(Intercept) | 7.84963 | 0.30929 | 25.3795 | ***0.001*** |
