# Supplementary material for: Guest-ant social parasites avoid conflict with social hosts using venom signaling
Source: PLoS One. 2026 Aug 12;21(8):e0345143. doi: 10.1371/journal.pone.0345143 (PMC13465834; doi:10.1371/journal.pone.0345143)
Supplement: Supplementary file 4 [file pone.0345143.s005.docx]

**S5 Appendix. Summary of interaction termination**.

**S5 Fig. Summary of interaction termination**. Each plot shows a hierarchical summary of behavioral conditions occurring prior to termination, partitioned by the actor which terminated. The sample size for each sunburst plot is shown to its left. Within a plot, each ring depicts a different behavioral component of conflict interactions (presence/absence) which are ordered from innermost to outermost according to a stereotypical interaction sequence. Rings are hierarchically arranged (clockwise), first by host aggression, then parasite venom, then penultimate behavior within each interaction. The inner core depicts the proportion of interactions in which biting occurred (no bite vs. bite). The second layer depicts venom escalation fraction (no venom, indirect, direct) in shades of brown, arranged by bite conditions in ring 1. The middle rings depict *presence/absence* for one of three host submissive behaviors (antenna tuck, back up, turn; rings 3-5 respectively) within a particular interaction. Lastly, the outer ring shows the specific conflict behavior (host or parasite) directly preceding interaction termination (arranged by bite and venom condition; layers 1 & 2). Host behaviors are shown in cool tones (purples, blues), while parasite venom behaviors are shown as warm tones (oranges, reds). “Other” denotes behaviors that are not covered in the ethogram or were unclear during video scoring. Four interactions were excluded because the end of the scoring window or video recording was reached before interaction termination occurred.

**S5 Text. Results**

Venom use (indirect, direct), co-occurred with some form of host submission behavior in 20 out of 23 interactions where venom was used; including 5 out of 6 interactions where indirect venom was present where hosts did not escalate to biting. However, parasites also terminated in 21 out of 23 interactions where venom was used, and always terminated whenever direct stinging occurred (9 interactions, 5 excluding mutual termination).

While host submissive behavior was not clearly related to termination by a particular actor, specific submissive behaviors did not always co-occur within interactions. For instance, host antenna tuck (layer 3) occurred most often in interactions where venom was present and parasites chose to terminate the interaction (11/19 interactions, or 9/14 excluding mutual termination, vs 1/9 interactions). While host turning (layer 5) occurred in nearly all interactions that hosts terminate where venom has been used (7/8 interactions, or 2/2 excluding mutual termination). These data suggest that specific submissive host behaviors may be used under different conflict contexts.
